# Comparison of outcomes and characteristics of patients admitted to the ICU with COVID-19 and other community-acquired pneumonia based on propensity score matching

**DOI:** 10.1186/s12879-024-09306-z

**Published:** 2024-04-22

**Authors:** Hongli Zhao, Xiulin Yan, Ziru Guo, Kaiyu Li, Zhaopeng Wang, Jun Wang, Dong Lv, Jianling Zhu, Ye Chen

**Affiliations:** 1 Department of Critical Care Medicine, Datong Third People’s Hospital, Datong, Shanxi China; 2 Science and Education Section, Datong Third People’s Hospital, Datong, Shanxi China

**Keywords:** COVID-19, Community-acquired pneumonia, Propensity score matching

## Abstract

**Objective:**

To compare the similarities and differences between patients with Coronavirus Disease 2019 (COVID-19) and those with other community-acquired pneumonia (CAP) admitted to the intensive care unit (ICU), utilizing propensity score matching (PSM), regarding hospitalization expenses, treatment options, and prognostic outcomes, aiming to inform the diagnosis and treatment of COVID-19.

**Methods:**

Patients admitted to the ICU of the Third People’s Hospital of Datong City, diagnosed with COVID-19 from December 2022 to February 2023, constituted the observation group, while those with other CAP admitted from January to November 2022 formed the control group. Basic information, clinical data at admission, and time from symptom onset to admission were matched using PSM.

**Results:**

A total of 70 patients were included in the COVID-19 group and 119 in the CAP group. The patients were matched by the propensity matching method, and 37 patients were included in each of the last two groups. After matching, COVID-19 had a higher failure rate than CAP, but the difference was not statistically significant (73% vs. 51%, *p* = 0.055). The utilization rate of antiviral drugs (40% vs. 11%, *p* = 0.003), γ-globulin (19% vs. 0%, *p* = 0.011) and prone position ventilation (PPV) (27% vs. 0%, *p* < 0.001) in patients with COVID-19 were higher than those in the CAP, and the differences were statistically significant. The total hospitalization cost of COVID-19 patients was lower than that of CAP patients, and the difference was statistically significant (27889.5 vs. 50175.9, *p* = 0.007). The hospital stay for COVID-19 patients was shorter than for CAP patients, but the difference was not statistically significant (10.9 vs. 16.6, *p* = 0.071).

**Conclusion:**

Our findings suggest that limited medical resources influenced patient outcomes during the COVID-19 pandemic. Addressing substantial demands for ICU capacity and medications during this period could have potentially reduced the mortality rate among COVID-19 patients.

## Objective

Since November 2019, the rapid outbreak of acute infectious Coronavirus Disease 2019 (COVID-19) caused by severe acute respiratory syndrome coronavirus type 2 infection has become a public health emergency of international concern [1]. Clinical symptoms of patients with COVID-19 are mostly fever, cough, sore throat and malaise, accompanied by lymphopenia and characteristic CT manifestations [2–3].The literature [4] suggests that approximately 80% of COVID-19 patients have mild symptoms and a good prognosis, while the remaining 20% may develop severe symptoms such as arterial hypoxaemia and respiratory distress. Critical cases may result in acute respiratory distress syndrome, multi-organ failure, and septic shock [5]. During the early stages of the pandemic, the mortality rate for severe COVID-19 patients was reported to be as high as 40% [6–9]. Community-acquired pneumonia (CAP) is the most common infectious disease of the respiratory system, affecting all ages, and is a major health problem. CAP has plagued humans for centuries, and worldwide, CAP affects 3 to 4 million people each year [10]. In European and American countries, CAP is the main cause of hospitalization and death of patients, and patients need to bear expensive medical expenses [11], resulting in huge clinical and economic burden [12]. In clinical diagnosis and treatment, it has been observed that patients with COVID-19 exhibit similar clinical symptoms and features to those with other CAP. Given the high morbidity and mortality of both COVID-19 and CAP, and the large number of cases of infection resulting from the rapid spread of COVID-19 in the community during the COVID-19 pandemic, it is critical to distinguish between COVID-19 pneumonia and SARS-CoV-2-negative CAP.

Due to significant variations in patient populations and healthcare resources among different countries, there is a lack of standardized reporting on the mortality rate of COVID-19 patients internationally [13–14]. This has led to substantial differences in the outcomes, indicators, and characteristics observed in various studies comparing COVID-19 with CAP [15–17]. Higgins TL et al. [18] showed that mortality and length of stay in patients with COVID-19 were higher than in other patients with CAP admitted to intensive care unit (ICU) in the United States. Wilde et al. [19] showed that lack of ICU beds resulted in increased mortality of COVID-19 patients. A German study [20] suggested that the higher mortality rate among COVID-19 patients was not due to insufficient medical resources, as the German health system did not reach its limits during the COVID-19 pandemic. The study utilized the propensity score matching (PSM) method to compare the mortality and clinical characteristics of two patient cohorts, aiming to mitigate the influence of potential confounding factors on patient mortality. This approach can be challenging to implement using conventional research methodologies.

PSM refers to the screening of the experimental and control groups using specific statistical methods to ensure that the selected subjects are comparable in terms of clinical characteristics. This ensures that any differences in outcomes between the two groups can be attributed solely to exposure factors. PSM serves to mitigate the influence of confounding variables and eliminate interference factors between groups [21].

In this study, the PSM method helps mitigate potential increases in COVID-19 mortality resulting from confounding factors such as inadequate medical resources and patient characteristics. Additionally, by comparing clinical data from COVID-19 and CAP patients, the similarities and differences in hospitalization cost, treatment plan and prognosis outcome were analyzed, and whether COVID-19 and CAP can learn from each other in treatment was discussed, hoping to guide the diagnosis and treatment of COVID-19 and CAP. It provides a reference for improving treatment measures, reasonably controlling hospital expenses, and reducing the economic burden of the country.

## Methods

### Study subjects

Patients with COVID-19 admitted to the ICU of the Third People’s Hospital of Datong City from December 2022 to February 2023 were selected as the observation group, and patients with other CAP admitted to the ICU of the Third People’s Hospital of Datong City from January to November 2022 were selected as the control group. Inclusion criteria: diagnosis of COVID-19 or CAP; age ≥ 18 years. Exclusion criteria: pregnant women; incomplete case data.

### Observational indicators

General information, time from symptom onset to consultation, history of chronic underlying disease (diabetes, hypertension, and lung diseases, especially underlying diseases such as COPD, affect lung capacity), whether or not the ventilator was used, APACHE II score, laboratory tests, pathogenetic and imaging results, treatment plan, ICU hospitalization time, cost and prognosis were collected from all patients who met the inclusion criteria. General information included gender and age. Laboratory examination included oxygen saturation, oxygenation index, blood urea nitrogen, white blood cell count, platelet count, neutrophil count and percentage, lymphocyte count and percentage, neutrophil/lymphocyte ratio, hemoglobin, hematocrit, alanine aminotransferase, aspartate aminotransferase, total bilirubin, blood creatinine, albumin, blood glucose, alkaline phosphatase, γ-glutamyl transferase, lactate dehydrogenase, prothrombin time, activated partial thromboplastin time, and D-dimer. Treatment regimens included the use of antimicrobials, antiviral therapy, γ-globulin, prone position ventilation (PPV), glucocorticoids, fluid resuscitation, and continuous renal replacement therapy.

### Outcomes

The primary outcome measure was the failure rate in both groups, and the secondary outcomes measure were the use rate of various treatments, length of hospital stay and hospitalization cost in both groups.

### Propensity score matching

The basic information of the patients was matched by PSM, using 1:1 nearest neighbor matching algorithm with a caliper value of 0.2. Matching information included the patient’s age, sex, time from symptom onset to consultation, APACHE score, temperature, respiration rate, pulse rate, mean arterial pressure, oxygen saturation, oxygenation index, blood urea nitrogen, white blood cell count, platelet count, neutrophil count and percentage, lymphocyte count and percentage, neutrophil to lymphocyte ratio, hemoglobin, hematocrit, alanine aminotransferase, aspartate aminotransferase, total bilirubin, creatinine, albumin, blood glucose, alkaline phosphatase, γ-glutamyltransferase, lactate dehydrogenase, prothrombin time, activated partial thromboplastin time, D-dimer, hypertension, diabetes, respiratory illnesses.

### Statistical analysis

Data were analyzed using SPSS26.0 statistical software. Measured data that conformed to normal distribution were expressed as mean ± standard deviation (x ± s); non-normally distributed data were expressed as median (quartile) [M (QL, QU)], and comparisons between groups were made using the independent use of independent samples t-test or Manner-Whitney rank-sum test. Count data were expressed as percentages, and comparisons between groups were made using the χ2 test or Fisher’s exact probability method. Outcome and treatment regimen analyses were performed using the chi-square test, and length of hospitalization and expenses during hospitalization were analyzed using analysis of variance (ANOVA). *p* < 0.05 was considered a statistically significant difference.

## Results

### Baseline information

In this study, 70 patients with COVID-19 and 119 patients with CAP were collected. The basic information and clinical data of the patients upon admission were matched at a 1:1 ratio using PSM. Finally, 37 patients with CAP and 37 patients with COVID-19 were included. Before matching, significant differences were observed between the two groups in terms of the time of symptom onset, oxygen saturation, white blood cell count, neutrophil count, lymphocyte ratio, and D-dimer levels. After matching, there were no significant differences between the two groups in terms of age, gender, time from symptom onset to hospitalization, temperature, respiratory rate and mean arterial pressure upon admission, laboratory results, and comorbidities. The differences between the two groups were not statistically significant. The above results indicated that the baseline characteristics of the two patient groups were balanced after PSM (Table 1).

**Table 1:** Characteristics of patients’ clinical information before and after propensity score matching

Characteristics	Unmatched			Matched		
	**CAP**, *N* = 119	**COVID-19**, *N* = 70	p-value	**CAP**, *N* = 37	**COVID-19**, *N* = 37	p-value
**Age (years)**			0.700			0.574
Median (IQR)	75 (66, 82)	76 (68, 83)		80 (70, 83)	78 (71, 83)	
**Sex**			0.081			0.812
Women	33 (28%)	28 (40%)		15 (41%)	14 (38%)	
Male	86 (72%)	42 (60%)		22 (59%)	23 (62%)	
**Time from symptom onset to hospitalization (days)**			0.013			0.180
Median (IQR)	5.0 (2.0, 7.0)	7.0 (4.0, 10.0)		4.0 (2.0, 7.0)	6.0 (4.0, 10.0)	
**APACHE IIscore**			0.017			0.953
Median (IQR)	21 (16, 27)	19 (14, 24)		19 (17, 25)	20 (15, 25)	
**Temperature (°C)**			0.254			0.575
Median (IQR)	36.60 (36.40, 37.00)	36.75 (36.50, 37.08)		36.60 (36.50, 37.10)	36.80 (36.50, 37.00)	
**Respiratory rate (BPM)**			< 0.001			0.744
Median (IQR)	25 (20, 30)	21 (20, 24)		22.0 (19.0, 25.0)	22.0 (20.0, 25.0)	
**Pulse rate (BPM)**			< 0.001			0.700
Mean ± SD	106 ± 23	95 ± 19		101 ± 24	99 ± 18	
**Mean arterial pressure (mmHg)**			0.036			0.168
Mean ± SD	89 ± 24	96 ± 19		91 ± 23	97 ± 15	
**Oxygen saturation (%)**			0.021			0.492
Median (IQR)	86 (76, 93)	89 (81, 96)		90 (83, 94)	85 (80, 96)	
**Oxygenation index**			0.287			0.304
Median (IQR)	188 (140, 251)	210 (153, 283)		235 (170, 279)	208 (124, 254)	
**Blood urea nitrogen (mmol/L)**			0.020			0.733
Median (IQR)	10 (6, 19)	7 (5, 14)		8 (5, 13)	9 (5, 17)	
**White blood cell count** (**10**^**9**^**/L**)			0.006			0.957
Median (IQR)	11 (7, 16)	9 (6, 14)		9.8 (7.1, 16.1)	10.3 (6.6, 15.4)	
**Platelet count** (**10**^**9**^**/L)**			0.222			0.462
Median (IQR)	198 (148, 238)	220 (154, 263)		204 (160, 236)	221 (147, 263)	
**Lymphocyte count** (**10**^**9**^**/L**)			0.600			0.592
Median (IQR)	0.80 (0.52, 1.27)	0.84 (0.57, 1.36)		0.93 (0.59, 1.19)	0.98 (0.72, 1.37)	
**Lymphocyte ratio (%)**			0.040			0.685
Median (IQR)	8 (4, 14)	11 (6, 16)		8.0 (5.3, 16.4)	10.4 (6.4, 15.8)	
**Neutrophil count** (**10**^**9**^**/L**)			0.004			0.935
Median (IQR)	9.8 (5.9, 13.9)	6.7 (4.3, 11.8)		7.8 (5.6, 14.0)	8.8 (4.9, 13.0)	
**Neutrophil ratio (%)**			0.038			0.405
Median (IQR)	86 (78, 91)	82 (74, 89)		86 (76, 90)	83 (75, 88)	
**Neutrophil /lymphocyte ratio**			0.030			0.646
Median (IQR)	11 (5, 20)	8 (4, 15)		11 (5, 17)	10 (5, 14)	
**Hemoglobin (g/L)**			0.923			0.717
Median (IQR)	131 (109, 150)	130 (112, 150)		130 (108, 141)	130 (111, 145)	
**Hematocrit (%)**			0.469			0.779
Median (IQR)	40 (33, 45)	39 (34, 44)		40 (33, 43)	39 (33, 43)	
**Alanine aminotransferase (U/L)**			0.133			0.581
Median (IQR)	24 (14, 40)	29 (17, 46)		30 (14, 35)	27 (17, 42)	
**Aspartate transaminase (U/L)**			0.687			0.816
Median (IQR)	33 (24, 58)	33 (21, 58)		30 (23, 47)	33 (21, 48)	
**Total bilirubin (umol/L)**			0.287			0.634
Median (IQR)	12 (8, 17)	11 (8, 14)		11 (6, 17)	10 (7, 14)	
**Creatinine (umol/L)**			0.536			0.037
Median (IQR)	78 (61, 159)	81 (53, 138)		64 (51, 84)	84 (59, 146)	
**Albumin (g/L)**			0.002			0.317
Median (IQR)	30 (27, 34)	33 (30, 35)		32.3 (28.7, 34.7)	32.8 (28.8, 35.5)	
**blood glucose (mmol/L)**			0.186			0.697
Median (IQR)	8.0 (6.6, 11.2)	9.2 (7.0, 11.7)		9 (7, 12)	9 (7, 12)	
**Alkaline phosphatase (U/L)**			0.894			0.358
Median (IQR)	89 (67, 118)	88 (65, 118)		88 (64, 109)	96 (73, 118)	
**γ-glutamyl transferase (U/L)**			0.020			0.897
Median (IQR)	29 (19, 55)	49 (23, 80)		36 (21, 53)	33 (21, 63)	
**Lactate dehydrogenase (U/L)**			0.005			0.093
Median (IQR)	297 (219, 434)	371 (280, 504)		278 (232, 401)	336 (279, 470)	
**Prothrombin time (sec)**			0.004			0.880
Median (IQR)	13.70 (12.70, 14.90)	12.85 (11.90, 14.23)		13.20 (12.30, 14.10)	13.20 (12.30, 14.80)	
**Activated partial thromboplastin time (sec)**			0.065			0.705
Median (IQR)	33 (30, 38)	32 (28, 35)		31 (28, 36)	32 (28, 37)	
**D-dimer (ug/L)**			0.023			0.996
Median (IQR)	3,230 (1,410, 7,745)	2,445 (710, 4,252)		2,250 (1,000, 3,390)	2,450 (710, 7,420)	
**Diabetes**			0.083			> 0.999
yes	27 (23%)	24 (34%)		12 (32%)	12 (32%)	
no	92 (77%)	46 (66%)		25 (68%)	25 (68%)	
**High blood pressure**			0.341			0.475
Yes	46 (39%)	32 (46%)		13 (35%)	16 (43%)	
No	73 (61%)	38 (54%)		24 (65%)	21 (57%)	
**Respiratory illnesses**			0.346			0.772
Yes	31 (26%)	14 (20%)		8 (22%)	7 (19%)	
No	88 (74%)	56 (80%)		29 (78%)	30 (81%)	

### Outcome and prognosis

As shown in Table 2, when the outcomes of the two groups were analyzed, the failure rate of patients with COVID-19 before matching was significantly higher than that of CAP (*P* < 0.05). The failure rate after matching was 73% for patients with COVID-19 and 51% for CAP, and the difference was not statistically significant.

**Table 2:** Comparison of Prognostic Outcome Indicators for COVID-19 and CAP Before and After Propensity score Matching

Characteristics	Unmatched			Matched		
	**CAP**, *N* = 119	**COVID-19**, *N* = 70	p-value	**CAP**, *N* = 37	**COVID-19**, *N* = 37	p-value
**Outcome**			0.020			0.055
Improved	58 (49%)	22 (31%)		18 (49%)	10 (27%)	
No improved	61 (51%)	48 (69%)		19 (51%)	27 (73%)	

### Treatment plan

The proportion of patients with CAP using ventilator and antimicrobial drugs was significantly higher than that of patients with COVID-19 before matching, and there was no significant difference between the two groups after matching; however, the proportions of patients with COVID-19 group in antiviral therapy, γ-globulin, and PPV were higher than that of the CAP group both before and after matching, and the difference was statistically significant (*P* < 0.05). Other therapeutic regimens such as glucocorticoids, fluid resuscitation, and anticoagulation therapy were not significantly different between the two groups of patients before and after matching (Table 3).

**Table 3:** Comparison of treatment regimens for COVID-19 and CAP before and after propensity matching

Characteristics	Unmatched			Matched		
	**CAP**, *N* = 119	**COVID-19**, *N* = 70	p-value	**CAP**, *N* = 37	**COVID-19**, *N* = 37	p-value
**Use of ventilators**			0.005			0.80
Yes	98 (82%)	45 (64%)		27 (73%)	26 (70%)	
No	21 (18%)	25 (36%)		10 (27%)	11 (30%)	
**Antimicrobial**			0.027			0.24
Yes	118 (99%)	65 (93%)		37 (100%)	34 (92%)	
No	1 (0.8%)	5 (7.1%)		0 (0%)	3 (8%)	
**Antiviral therapy**			< 0.001			0.003
Yes	13 (11%)	28 (40%)		4 (11%)	15 (41%)	
No	106 (89%)	42 (60%)		33 (89%)	22 (59%)	
**γ-globulin**			< 0.001			0.011
Yes	2 (1.7%)	13 (19%)		0 (0%)	7 (19%)	
No	117 (98%)	57 (81%)		37 (100%)	30 (81%)	
**Prone position ventilation**			< 0.001			< 0.001
Yes	0 (0%)	21 (30%)		0 (0%)	10 (27%)	
No	119 (100%)	49 (70%)		37 (100%)	27 (73%)	
**Glucocorticosteroid**			0.24			0.64
Yes	49 (41%)	35 (50%)		16 (43%)	14 (38%)	
No	70 (59%)	35 (50%)		21 (57%)	23(62%)	
**Fluid resuscitation**			0.057			0.62
Yes	43 (36%)	16 (23%)		13 (35%)	11 (30%)	
No	76 (64%)	54 (77%)		24 (65%)	26 (70%)	
**Continuous renal replacement therapy**			0.59			>0.99
Yes	15 (13%)	7 (10%)		5 (14%)	4 (11%)	
No	104 (87%)	63 (90%)		32 (86%)	33 (89%)	
**Anticoagulation**			0.79			0.16
Yes	52 (44%)	32 (46%)		18 (49%)	12 (32%)	
No	67 (56%)	38 (54%)		19 (51%)	25 (68%)	
**Vasopressors**			0.27			0.24
Yes	76 (64%)	39 (56%)		18 (49%)	23 (62%)	
No	43 (36%)	31 (44%)		19 (51%)	14 (38%)	

### Length of hospitalization and costs

ANOVA was done on the data before and after matching, the total hospitalization time and ICU stay were greater in the CAP group than in the COVID-19 group, with no statistically significant difference (*P* > 0.05), and the total cost, total drug cost, test cost, examination cost and treatment cost were greater in the CAP group than in the COVID-19 group, with a significant difference (Table 4).

**Table 4:** Comparison of length of hospitalization and costs for COVID-19 and CAP before and after propensity matching

Characteristics	Unmatched			Matched		
	**CAP**, *N* = 119 (Mean(SD))	COVID-19 *N* = 70 (Mean(SD))	p-value	**CAP**, *N* = 37 (Mean(SD))	**COVID-19**, *N* = 37 (Mean(SD))	p-value
Total length of stay (days)	14.2 (13.01)	12.4 (9.52)	0.333	16.6 (15.91)	10.9 (10.26)	0.071
Length of ICU stay (days)	10.4 (8.64)	8.8 (7.67)	0.215	11.5 (8.95)	8.1 (8.56)	0.096
Total costs (¥)	48385.1 (43946.88)	29356.8 (29409.72)	0.002	50175.9 (38296.32)	27998.5 (30079.56)	0.007
Average daily cost (¥)	4336.6 (2530.91)	2795.2 (1995.68)	< 0.001	3908.6 (2438.08)	3171.2 (2029.21)	0.162
Total drug costs (¥)	14473.6 (18995.99)	6388.4 (8544.39)	< 0.001	14703.4 (14086.47)	5940.8 (9152.08)	0.002
Laboratory fee (¥)	4893.7 (3286.91)	3196.0 (2504.27)	< 0.001	5272.7 (3673.55)	3067.9 (2472.38)	0.003
Inspection fee (¥)	736.0 (686.7)	367.4 (364.14)	< 0.001	868.7 (798.91)	350.9 (344.83)	< 0.001
Treatment cost (¥)	10216.6 (9615.23)	7092.1 (8403.64)	0.025	11113.3 (8953.29)	6863.7 (8469.33)	0.039

## Discussion

The COVID-19 pandemic, caused by SARS-CoV-2, has led to a sudden and large increase in the number of hospitalizations worldwide for pneumonia combined with multi-organ disease. The overall in-hospital mortality rate for COVID-19 is about 15–20%, but as high as 40% in patients requiring admission to the ICU [7]. However, mortality rates varied from cohort to cohort. A meta-analysis [22] suggested that such a wide range of differences may be due to national conditions and basic patient conditions in different countries. CAP has long plagued mankind because of its high morbidity and mortality. In the current outbreak, people have tried to compare death rates from the two diseases, and because there is no standardized reporting for COVID-19, the results of the comparison are not reliable. In this study, PSM is used to control confounding factors that may affect mortality, and the outcomes of COVID-19 patients and CAP patients are compared, and the results showed no difference in mortality between the two patients. Moreover, through the analysis of clinical data such as treatment measures, length of stay and hospitalization cost, the shortage of medical resources during the pandemic contributed to the high mortality rate of COVID-19.

The study revealed that the failure rate among COVID-19 patients prior to matching was 69%, significantly higher than that of CAP. Moreover, the duration from symptom onset to admission was significantly longer for COVID-19 patients compared to those with CAP. After matching for confounding factors such as the duration from symptom onset to admission, baseline admission information, and laboratory indices, the failure rate of COVID-19 remained at 73%, showing no statistically significant difference from that of CAP. Therefore, the increased strain on hospital beds and the heightened workload for medical personnel during the COVID-19 pandemic may have had detrimental effects on patient.

In the course of treatment, patients with COVID-19 exhibit a higher utilization rate of antiviral therapy, γ-globulin and PPV compared to patients with CAP. According to the Diagnostic and Treatment Protocol for COVID-19 Infection (Trial Ninth Edition) [23], antiviral drugs and intravenous human immunoglobulin should be administered to all patients with high-risk factors, high viral loads, and rapid disease progression. However, the utilization rate of antiviral drugs was 40% for patients with COVID-19 before matching and 41% after matching, with more than half of the patients not receiving antiviral treatment. The utilization rate of γ-globulin remained at 19% both before and after matching. Thus, the shortage of medical resources may also impact the prognosis of patients with COVID-19.

It can be inferred from past experience that diseases with complex conditions and high mortality rates are associated with extended hospital stays and elevated costs. However, regardless of whether the PSM is used, the cost of COVID-19 patients is lower than that of CAP, and the length of hospital stay is not different between the two. A reasonable guess is that this result is due to a shortage of medical resources due to the COVID-19 outbreak, with many medications and tests being replaced by free treatments such as prone ventilation. On the other hand, due to the shortage of medical resources, many patients did not receive timely treatment and their condition had progressed to an irreversible point when they were admitted to hospital. As a result, the hospital stay of patients with COVID-19 was not longer than that of patients with CAP. Some patients were discharged due to deterioration of their condition, so the mortality rate of patients without PSM was higher.

In summary, through the retrospective study of patients with severe COVID-19 and patients with severe CAP, it was found that by eliminating the effects of some confounding factors such as the time from the appearance of symptoms to the time of medical consultation, the mortality rate of patients with COVID-19 was not significantly higher than that of patients with CAP. Therefore, medical resources can be strengthened, standardized operating procedures and methods can be developed to improve medical efficiency, and through health education to improve patients’ awareness of timely medical treatment when relevant symptoms appear.

However, our study still has certain limitations. As it is a retrospective study, the time span for collecting cases of community-acquired pneumonia is broad, introducing the possibility of bias. Additionally, all patients are from a single research unit in our hospital, resulting in a small sample size. In addition this study should be further subgroup analysis to compare whether there are differences in treatment measures, cost and hospitalization days between the two groups of patients with different prognosis, but due to the small number of cases, it is not reliable to carry out subgroup analysis of the outcome. This study is only a starting point, and hopefully, more researchers can provide relevant studies to make up for the shortcomings of this study in the future.

## Conclusion

Shortages of hospital beds and drug supplies during the COVID-19 pandemic increased the failure rate for COVID-19, making it even higher than that for CAP. Our research shows that after removing these factors, the failure rates are the same. Therefore, addressing healthcare demands could potentially reduce the mortality rate among COVID-19 patients. However, more research is needed to further confirm these findings.

In conclusion, our study offers valuable insights for shaping future public health policies and healthcare system planning. Enhanced preparedness and responsiveness are pivotal in bolstering our capacity to combat pandemics, thereby safeguarding the lives and well-being of individuals worldwide.

## Data Availability

The datasets used and/or analysed during the current study available from the corresponding author on reasonable request.

## Abbreviations

COVID-19: Corona Virus Disease 2019
CAP: community acquired pneumonia
ICU: intensive care unit
PSM: propensity score matching
PPV: prone position ventilation
ANOVA: anslysis of variance
